# Health inequality in adolescence. Does stratification occur by familial social background, family affluence, or personal social position?

**DOI:** 10.1186/1471-2458-6-110

**Published:** 2006-04-27

**Authors:** Koivusilta LK, Rimpelä AH, Kautiainen SM

**Affiliations:** 1 Department of Social Policy and IASM (Institutions and Social Mechanisms), FIN-20014 University of Turku, Finland; 2 School of Public Health, FIN-33014 University of Tampere, Finland

## Abstract

**Background:**

Two new sets of stratification indicators – family's material affluence and adolescent's personal social position- were compared with traditional indicators of familial social position based on parental occupation and education for their ability to detect health inequality among adolescents.

**Methods:**

Survey data were collected in the Adolescent Health and Lifestyle Survey in 2003 from nationally representative samples of 12-, 14- and 16-year-old Finns (number of respondents 5394, response rate 71%). Indicators of the familial social position were father's socio-economic status, parents' education, parents' labour market position. Indicators of material affluence were number of cars, vacation travels, and computers in the family, own room and amount of weekly spending money. Adolescent's personal social position was measured as school performance. Measures of health were long-standing illness, overweight, use of mental health services, poor self-rated health and number of weekly health complaints. Ordinal logistic regression analysis was applied to study the associations between stratification indicators and health variables.

**Results:**

All three groups of indicators of social stratification showed inequality in health, but the strongest associations were observed with the adolescent's personal social position. Health inequality was only partly identifiable by the traditional indicators of familial social position. The direction of the inequality was as expected when using the traditional indicators or personal social position: adolescents from higher social positions were healthier than those from lower positions. The indicators of family's material affluence showed mainly weak or no association with health and some of the indicators were inversely associated, although weakly.

**Conclusion:**

In addition to traditional indicators describing the socio-structural influences on the distribution of health among adolescents, indicators of family's material affluence should be further developed. Adolescents' personal social position should be included in the studies of health inequalities.

## Background

According to the prevailing understanding, the phenomenon of lower social strata suffering from worse health as compared to upper strata is less uniformly visible in adolescence than in childhood or adulthood. Instead, there seems to exist a health equality, or at least a variation in health differences depending on the indicators used for health and social position [1,2]. Health differences re-emerge, however, as soon as young people reach adulthood and enter the labour market [3]. Thus, there is cogent reason to suppose that also young age groups are touched by some health inequality.

The results of health distribution in adolescence mainly derive from traditional stratification indicators that measure social class by father's or other guardian's occupational status and/or educational level. The obscurity of health differences has been attributed to the inappropriate indicators at this stage of life [4]. Social stratification may be understood as a single phenomenon or as several phenomena. The starting point for our analysis is that inequality between population groups can be approached from various perspectives. Indicators of health-related social stratification in adolescence may be based on various spheres of life relevant for health and well-being of young age groups. While the traditional indicators of social position reflect the social-structural basis of living conditions, these indicators differ in their sensitivity in catching health inequality during the adolescent years. Thus, other kinds of indicators may be more efficient in measuring social inequality, which indeed exists between young people.

Our interest here is the explanation related to a possible inadequacy of traditional indicators of social position in adolescence. Can father's or other guardian's occupational status and educational level measure social position at the time when a young person is experiencing a transition from being a child living with parents' care to a more independent actor in a wider society. During this developmental course, multiple and simultaneous transitions and challenges in various spheres of life may increase the risk for poor health [5]. Optional indicators can be sought from two sources: adolescent's personal social position as defined through schooling and education and family's possession of material commodities important for adolescence as reflecting the standard of living.

Earlier studies have shown that factors related to the formation of a young person's own social status through choices and success in educational career play a role in producing health inequality [6]. For adolescents committed to attaining educational goals, school represents a neighbourhood society with favourable social networks. These, in turn, influence health positively [7]. The social, cultural and intellectual resources provided by education also contribute to health-promoting choices [8]. There is evidence that adolescents, who discontinue education after comprehensive school commonly engage in various health-compromising behaviours [6], typical of lower socio-economic strata [9].

A new question battery, the Family Affluence Scale (FAS), was originally based on the work of Peter Townsend [10]. It rests on the usefulness of other than occupation-based indicators for social standing, and was further developed by the Health Behaviour in School-Aged Children Study (HBSC). Its item questions deal with the possession of various commodities, like equipment and objects reflecting the level of material well-being in families [11]. Use of these indicators is believed to reduce the problems involved in using the traditional indicators of familial social position in adolescents, such as non-response caused by difficulty in answering questions on parents' socio-economic position. In particular, young respondents whose parents are not gainfully employed at the moment of inquiry may remain uncovered, or the precise positions of the parents may remain open for interpretation. Furthermore, there is no satisfactory way of differentiating between parents with a social status like student, housewife or husband, active job seeker, or retired. Ascertaining the degree to which the parent not living at home contributes to family's financing is equally uncertain. In reconstituted families, it is not obvious whose occupational status is the most relevant [11]. Absent or inappropriate response often leads to low validity of the study [12,13].

Our objective is to study three groups of indicators of social stratification in terms of how they classify adolescents according to various dimensions of health. Besides the traditional indicators of familial social position (father's or other guardian's occupation and education) we use indicators based on family affluence (material commodities) and one indicator based on adolescent's own social position measured by school performance. In addition, the traditional indicators are complemented with mother's education and labour market position of both parents. The material is representative of Finland.

## Methods

### Participants

Data were collected in the Adolescent Health and Lifestyle Survey between February and April 2003 from nationally representative samples based on dates of birth of 12-, 14- and 16-year-old Finns using a self-administered structured mailed questionnaire with two re-inquiries to non-respondents. The sample was drawn from the Population Register Centre by selecting all Finns born at certain adjacent dates in July. The total sample size was 7648 and the number of respondents was 5394. The response rates were somewhat higher in girls than in boys. Among 12-, 14- and 16-year-old boys, 69%, 66%, and 59% returned the questionnaires, and among girls, 75%, 78% and 79%, respectively. No consent was needed according to the Finnish legislation to collect and analyze these questionnaire data. The participants knew that responding was voluntary and that the questionnaire was confidential. The ethical committee of the Department of Public Health at the University of Helsinki accepted the study protocol.

### Health measures

Health was measured as

• Long-standing illness or disability restricting daily activities, or continuous medication prescribed by doctor at the time of inquiry: no, yes.

• Overweight. Based on self-reported weight and height, overweight and obesity were determined using the international age-specific cut-off points [14] for body mass index (weight in kilograms divided by height in meters squared). The categories were: normal weight, overweight, obesity.

• A rough measure of mental health problems was attained by asking about the use of mental health services (e.g. outpatient clinic of child and adolescent psychiatry, family counselling centre, psychologist or psychiatrist) during the preceding two years: no, yes.

• Self-rated health at the time of inquiry: very good, good, moderate/poor.

• Weekly health complaints were investigated by constructing a sum-index on the frequency of feeling daily, during the preceding 6 months, any of eight health complaints listed in the questionnaire (stomach aches, tension or nervousness, irritability or outbursts of anger, trouble falling asleep or waking at night, headache, trembling of hands, feeling tired or weak, feeling dizzy): 0, 1, 2, 3 or more.

### Indicators of social stratification

Familial social position was measured by

• Father's or other guardian's socio-economic status (SES) was encoded from open-ended responses to a question of the primary profession, position or activity according to the Status Classification of Statistics Finland [15]: upper white-collar employee, entrepreneur, lower white-collar employee, blue-collar employee or other (i.e. unclassified);

• Parent's educational level: either one has high level (12 years or more in education), either one has middle level (approximately 9–11 years), both have low level (approximately 9 years or less). Father's and mother's education were asked separately.

• Parent's labour market position: both are gainfully employed (outside or at home), at most one parent is gainfully employed (the other being unemployed or laid-off, retired or on extended/long-lasting sick leave). Father's and mother's labour market position were asked separately.

Family affluence was measured by

• Number of cars in family: 0, 1, 2 or more.

• Number of family's vacation travels during the last year: 0, 1, 2, 3 or more.

• Number of computers in household: 0 1, 2, 3 or more.

• Asking if the respondent occupied a room of one's own: yes, no.

• Amount of weekly spending money (not including housing, food and clothing expenses): four categories based on distribution categories calculated separately for each combination of age and gender.

Adolescent's personal social position.

• Personal social position was measured by school achievement. In the questionnaire, the pupil was asked to assess his/her position in class according to preceding end-of-term school report. The categories were: much above average, slightly above average, average, below average. By age 16, Finnish adolescents have made the decision whether to continue education after the compulsory phase. Discontinuing education strongly anticipates poor social position in adulthood [16]. Thus, adolescents not in education or those still in comprehensive school at age 16 were classified in the category "below average".

### Statistical methods

First, associations (MODEL 1) between stratification indicators and health measures were studied using ordinal logistic regression analysis which allows modelling of a polytomous ordinal response on set of predictors [17]. Second, analyses were performed to study which indicators within each group of stratification indicators were independently associated with each health variable (MODEL 2). Third, variables showing independent associations within each set of stratification indicators were further included in the final multivariate analyses to yield models of independent indicators for each health variable (FINAL MODEL). Cumulative odds ratios with 95% confidence intervals were calculated, and, throughout, gender- and age-adjusted results were given. The associations in MODELS 1 were also studied separately in boys and girls. Due to a strong relationship of personal social position with age and gender, the associations between personal social position and health variables were also studied according to gender in age groups 12/14 and 16. The categories giving approximately equal odds ratios were combined in MODELS 2 and the FINAL MODELS. A p value of <0.05 was used as the cut-off point of statistical significance throughout the study. Analyses were performed using the Statistical Package for Social Sciences (SPSS 12.0 for Windows).

## Results

Table 1 shows the distributions of the variables in the total material. There were more missing values in the traditional indicators than in the new type of indicators. The proportion of missing values was highest for father's SES (5%). The proportions of missing values were high in questions measuring father's and mother's education and labour market position separately, but the proportions were smaller for variables combining the information of both parents.

### Health differences according to stratification indicators

#### Familial social position

Father's low SES was indicative of overweight, use of mental health services and poor self-rated health among adolescents (MODEL 1; Table 2). The child had a higher risk of being overweight or having poor self-rated health if both parents had at most middle education level compared to at least one parent having a high education level. Both parents' low level of education was suggestive of the adolescent's long-standing illness, again compared to at least one parent having a high education level. Adolescents with at most one parent participating in the labour force were more often overweight, used mental health services, had poorer self-rated health and more weekly health complaints than adolescents with both parents gainfully employed.

Some of the associations existed only in either gender. Parents' labour market position was associated with overweight and weekly health complaints in girls only, and father's SES was associated with self-rated health and weekly health complaints in girls only. Parents' education in turn was associated with the use of mental health services in boys only.

Parents' labour market position and education retained their positions as independent predictors of overweight and self-rated health, while SES lost its statistical significance (MODEL 2). Father's SES and parents' labour market position emerged as independent predictors of the use of mental health services.

#### Family affluence

The number of cars in the family was slightly associated with adolescent's use of mental health services (MODEL 1; Table 3). The association with overweight was clearer: overweight of adolescent was more likely in families not owning a car compared to families owning at least two cars. Less vacation travels indicated worse adolescent's self-rated health. Further, compared to at least two vacation travels, no vacation travels indicated use of mental health services, overweight, and several health complaints. The computer variable was weakly but statistically significantly associated with self-rated health. Compared to the highest quartile of weekly spending money, the second lowest quartile was indicative of a lower probability of long-standing illness and of non-use of mental health services. Adolescents in the lowest category of weekly spending money were overweight less often than those having the most spending money. Adolescents with or without a room of their own showed no differences in health.

Some of the associations were found in one gender only. The associations between the number of cars and overweight, between number of vacation travels and long-standing illness and weekly health complaints, and between a room of one's own and weekly health complaints, were all found in girls only. A room of one's own implicated more health complaints. The amount of weekly spending money was associated with long-standing illness, use of mental health problems, poorer self-rated health and weekly health complaints among girls, but, in boys, the amount of weekly spending money was associated with overweight.

All variables showing association with various health variables in MODELS 1 were independent predictors of health as well (MODELS 2).

#### Adolescent's personal social position

School achievement was statistically significantly associated with each health measure, except long-standing illness. Worse school achievement indicated an increased risk of experiencing poor health (MODELS 1–2; Table 4). However, there was an association between long-standing illness and achievement among girls: all the other achievement groups differed statistically significantly from the best achieving girls in their higher risk of being chronically ill.

Associations between personal social position and health variables were also studied separately in boys aged 12/14, girls aged 12/14, boys aged 16, and girls aged 16. In the analyses for the 16-year-olds, those who discontinued school or who still were in comprehensive school were omitted. There were no associations between personal social position and long-term illness in any subgroup. Differences in overweight according to personal social position were seen in girls, but not in boys. Associations with the use of mental health services resembled those in the whole material, with the exception of 16-year-old boys, for whom no statistically significant associations existed. Associations with self-rated health were comparable to those calculated in the whole material, except for boys: the two highest achieving groups did not differ from each other in either age group. The detailed study of weekly health complaints showed that the best achieving students and those with average school marks differed statistically significantly among the youngest girls only.

#### Final multivariate analyses

Many of the associations observed at the foregoing steps of the analyses lost their statistical significance in the final multivariate models, consisting of independent predictors from each group of stratification indicators (FINAL MODEL, Table 5). Among the traditional indicators of familial social position, parents' labour market position remained as an independent predictor of every dimension of health, except of long-standing illness. Associations between personal social position and various health measures, except for long-standing illness, remained statistically significant.

## Discussion

A remarkable inequality seems to prevail in adolescent health. All three groups of indicators of social stratification showed inequality in health, but the strongest associations were observed with the adolescent's personal social position measured by school achievement. Health inequality was only partly identifiable by the traditional indicators of familial social position (father's SES, parents' education, parents' labour market position). The direction of the inequality was as expected for traditional indicators and in personal social position: adolescents from higher social positions were healthier than those from lower positions. The indicators of families' material affluence showed weak or no association with health and some of the indicators were inversely associated, although weakly.

Traditional indicators of familial social position reveal the structural basis for the families' economic and cultural resources, and thus constitute a way to follow the development and trends of living conditions of families with children. The influence of these indicators, however, varies during the years of adolescence [3,4]. Thus, they should not be relied on without considering other possible influences on adolescents' health in our modern and pluralistic societies.

We used five different indicators of familial material affluence: cars in the family, vacation travels, one's own room, computers in the household, and weekly spending money. The scale was adopted from previous studies [10,11]. The dimension of social position reflected by these affluence indicators may be debated. For example, vacation travelling not only contains economic components, but is also related with the quality of relationships between family members and the amount of time available to spend together. On the other hand, the number of computers may actually reflect other aspects of the adolescent's circumstances than those related to affluence, e.g. the nature of parent's occupation or the family's inclination towards information technologies. Affluent or highly educated parents may more readily provide their children with computers, but these social groups, like all the others, are not safe from experiencing the negative consequences of a computerized lifestyle (e.g. Internet addiction, daytime tiredness). The associations between health measures and the amount of weekly spending money were contrary to expectations. Spending money is influenced by, e.g., adolescents' participation in working life or the parenting style of the family, and it may thus, at least partly, measure something else than family affluence.

Cautiousness is called for, when using and developing indicators of family affluence. Some indicators may involve connotations such as closeness of family relations, amount of leisure, or contents of parents' work tasks, rather than reflect the families' material standard of living. The contexts formed by the trends and developmental processes in the larger society continuously influence the relevance and interpretation of the various indicators of family affluence. One example of this is the changing information and communication culture and its related equipment, and the society's pursuits to empower all citizens with Internet access, irrespective of their economic situation.

The adolescent's personal social position as indicated by school achievement was closely related with every health indicator, except for long-standing illness. The worse the school achievement, the bigger was the risk of experiencing poor health. The relationship was quite strong when evaluated in terms of mental health. A close relationship between adolescents' school performance and mental health was shown in a recent literature review as well [18]. Various kinds of mechanisms may constitute the underlying causes for this association. For example, there is evidence that school context may be related to adolescents' depressive symptoms [19], and that emotional or behavioural problems increase children's vulnerability to, e.g., poor classroom climate [20]. Supportive school life in turn has been found to act as a buffer against the adverse influence of poor family background [21].

Educational career and development of health resources have been found to be closely intertwined during one's life-course. Adolescents with poor health and several health-compromising behaviours in their lifestyle are at risk of reaching low educational levels in adulthood as compared to healthier adolescents and those with a more health-enhancing behavioural profile [6]. It is possible that in a society where education is highly valued, such as Finland, failure in school is a stressor for a young person. Poor achievement may thus gnaw self-confidence and lead to anxiety, which is measurable by health indicators [22].

School life is not separable from more fundamental societal factors structuring the economic options of families. It may simply be true that school matters are more fruitful in bringing out the inequality present in social structures. This became obvious in a Finnish study which, by analysing changes in regional and socio-economic health differences among youth from the 1980s to the beginning of the 21st century, indicated, that differences in youth health, according to conventional socio-economic factors were minimal. In contrast, factors dealing with school performance and educational careers were closely associated with indicators of youth welfare, suggesting that health differences will be likely in the future [23].

A wider range of indicators should be developed to identify such young people whose families lack the means or the resources to provide support for their educational careers. Whether indicating family affluence or whether applied for school and family contexts, the new indicators should follow the societal development and include functional items as well. Measuring the social capital constructed in families and other nearby environments like schools is an important line to follow in future research. The notion of relative deprivation could offer a fruitful starting point for the development of stratification indicators, because development in youth largely occurs through making comparisons with reference groups or "important others" [24].

Health differences between young people are not a direct or clear-cut result of economic inequality between families' social standings, but an outcome of several mechanisms. When problems accumulate and intertwine in these spheres of life, e.g. as a consequence of economic problems or inadequate social networks, health differences may begin to emerge and increase. This has been noticed in studies on social capital where causality has been ascertained to run from educational attainment to social capital, and vice versa as well [25].

The final multivariate analyses revealed a pattern of stratification largely occurring on grounds of parents' labour market position and the adolescent's school achievement. The study of stratification based on a division of indicators in some main groups may not offer the most fruitful starting point. Instead, investigators might focus on the synergy of indicators in accentuating or diminishing their impact on the various dimensions of health with a specific aim to determine the clusters or main dimensions of social stratification.

The proportion of missing values was higher in the traditional indicators of social position than in the other indicators. At least the youngest respondents were perhaps not able to choose the right options or to describe their parents' occupation or education. As remaining outside the labour force is more typical of people in the lower socio-economic strata, these variables summarize many health-relevant factors in families' life situations, economic factors inclusive. Accordingly, non-response conceals a proportion of health inequality that might become visible through other kinds of indicators. Furthermore, indicators based on parents' social position highlight the difficulty of identifying one growing group of adolescents, namely, those who are missing a parent or not having contact with the parent. The small amount of missing values for indicators of family affluence and school achievement suggests that other kinds of indicators could be used or developed to cover features of social environments relevant for adolescents' health.

To emphasize the essence, and for simplification, we have restricted the analysis to include age and gender mainly as adjustment factors. The bi-variate associations between each stratification indicator and each health indicator, calculated separately in boys and girls, showed that some associations exist among girls only. Evaluation of associations of personal social position measured by school achievement and health in age- and gender-specific groups showed that, despite some fluctuations, the associations were essentially similar in both sexes and at all ages.

Health is not a uniform phenomenon. To study how health differences develop and how they may be influenced, indicators from all the studied categories are applicable, but in-depth analysis on their points of reference is needed. A test for a potentially relevant indicator would measure whether the indicator identifies such features in the studied population that can conceivably be influenced or even changed. Indicators related to school have the advantage of dealing with factors belonging to adolescents' immediate environment and, consequently, they may also involve means for reducing health inequalities. The picture generated using a more colourful palette of stratification indicators may offer a wider assortment of measures to various fields and spheres of authority in their quest to prevent health differences among young people.

## Conclusion

The traditional indicators need to be used in describing the influence of socio-structural factors on the distribution of health among adolescents. In addition to these, indicators of family's material affluence should be further developed, e.g., to establish whether they contribute to adolescents' proneness to elect health-promoting lifestyles, like enabling acquisition of equipment for physical exercise. Adolescents' personal social position is a new indicator that should be included in the studies of health inequalities at that age group. Whether school achievement is a sufficient measure of personal social position, is the question to be answered by further studies.

## Competing interests

The author(s) declare that they have no competing interests.

## Authors' contributions

Arja Rimpelä was in charge of the data collection. Every author participated in the writing and review of drafts of the manuscript. Leena Koivusilta performed the statistical analyses. Every author read and approved the final manuscript.

## Pre-publication history

The pre-publication history for this paper can be accessed here:



## Figures and Tables

**Table 1:** Frequency and percentage distributions of respondents in categories of the study variables.

Variable	Distributions	
	N	%

*Familial social position*		

Father's SES		
Upper white collar	1241	23
Entrepreneur	725	13
Lower white collar	1217	23
Blue collar/Other	1933	36
Missing	278	5
		

Parent's education		
Either has high level	1018	19
Either has middle level	3506	65
Both have low level	734	14
Missing	136	2
		

Parent's labour market position		
Both gainfully employed	3918	73
At most one gainfully employed	1293	24
Missing	183	3
		

*Family affluence*		
		

Cars in the family		
Two or more	2389	44
One	1590	48
None	369	7
Missing	46	1
		

Vacation travels		
Three or more	1294	24
Two	1050	20
One	1527	28
None	1453	27
Missing	70	1
		

Computers		
Three or more	504	9
Two	1156	22
One	3117	58
None	440	8
Missing	177	3
		

Own room		
Yes	4706	87
No	640	12
Missing	48	1
		

Weekly spending money		
Above upper quartile	725	14
Median – Upper quartile	1690	31
Lower quartile – Median	1235	23
Below lower quartile	1641	30
Missing	103	2

*Personal social position*		

School achievement^1)^		
Much above average	762	14
Slightly above average	1658	31
Average	2178	40
Below average	739	14
Missing	57	1

*Health*		

Long-standing illness		
No	4574	85
Yes	760	14
Missing	60	1
		

Overweight		
Normal weight	4438	82
Overweight	653	12
Obesity	156	3
Missing	147	3
		

Use of mental health services		
No	4977	92
Yes	372	7
Missing	45	1
		

Self-rated health		
Very good	1749	32
Good	2670	50
Moderate or poor	935	17
Missing	40	1
		

Weekly health complaints		
0	2040	38
1	1230	23
2	782	14
3 or more	1342	25
Missing	0	0

Total number of respondents	n = 5394	100%

^1) ^Adolescents not in education or still in comprehensive school at age 16 (10% of boys and 9% of girls) were classified in the category "below average".

**Table 2:** Ordinal logistic regression models for associations between indicators of familial social position and poor health. Separate models for each health variable, adjusted for sex and age

	HEALTH INDICATOR				
STRATIFICATION INDICATOR	Long-standing illness	Overweight	Use of mental health services	Poor self-rated health	Weekly health complaints

	Cumulative odds ratios with 95% confidence intervals^2)^				

MODEL 1^1)^					
Father's SES					
Upper white collar	1.0 (ref)	1.0 (ref)	1.0 (ref)	1.0 (ref)	1.0 (ref)
Entrepreneur	1.0 (0.7–1.2)	**1.8 (1.4–2.3)**	0.7 (0.4–1.0)	1.1 (0.9–1.3)	1.1 (0.9–1.2)
Lower white collar	1.0 (0.8–1.2)	**1.3 (1.0–1.7)**	1.0 (0.7–1.4)	1.1 (0.9–1.3)	1.0 (0.9–1.2)
Blue collar/Other	0.9 (0.7–1.1)	**1.8 (1.4–2.2)**	**1.4 (1.0–1.8)**	**1.2 (1.1–1.4)**	1.1 (0.9–1.2)

Parent's education					
Either has high level	1.0 (ref)	1.0 (ref)	1.0 (ref)	1.0 (ref)	1.0 (ref)
Either has middle level	1.0 (0.8–1.2)	**1.6 (1.2–2.1)**	1.2 (0.9–1.6)	**1.2 (1.1–1.4)**	1.0 (0.9–1.1)
Both have low level	**1.4 (1.0–1.8)**	**2.1 (1.6–2.8)**	1.5 (1.0–2.1)	**1.4 (1.2–1.7)**	1.1 (0.9–1.3)

Parent's labour market position					
Both gainfully employed	1.0 (ref)	1.0 (ref)	1.0 (ref)	1.0 (ref)	1.0 (ref)
At most one gainfully employed	1.1 (0.9–1.3)	**1.4 (1.1–1.6)**	**1.9 (1.5–2.3)**	**1.4 (1.2–1.5)**	**1.2 (1.1–1.3)**

MODEL 2 ^3)4)^					

Father's SES	**. .**	*NS*		*NS*	**. .**
Higher			1.0 (ref)		
Blue collar/Other			**1.3 (1.0–1.6)**		

Parent's education			**. .**		**. .**
Either has high level	1.0 (ref)	1.0 (ref)		1.0 (ref)	
Either has middle level	1.0 (0.8–1.2)	**1.6 (1.3–2.6**)		**1.3 (1.1–1.5)**	
Both have low level	**1.4 (1.0–1.8)**	**2.0 (1.5–2.8)**		**1.3 (1.1–1.6)**	

Parent's labour market position	**. .**				
Both gainfully employed		1.0 (ref)	1.0 (ref)	1.0 (ref)	1.0 (ref)
At most one gainfully employed		**1.3 (1.0–1.5)**	**1.8 (1.4–2.2)**	**1.3 (1.2–1.5)**	**1.2 (1.1–1.3)**

^1) ^Models: Health variable = Stratification indicator + Sex/Age variable.

^2) ^Statistically significant cumulative odds ratios in bold.

^3) ^Models for stratification indicators independently associated with the health variable: Health variable = Stratification indicator_1 _+ Stratification indicator_2 _... +Stratification indicator_k _+ Sex/Age variable.

^4) ^The categories giving approximately equal odds ratios were combined.

**.. **= Not statistically significant in the previous step of analysis; *NS *= Not statistically significant in the model.

**Table 3:** Ordinal logistic regression models for associations between indicators of family affluence and poor health. Separate models for each health variable, adjusted for sex and age

	HEALTH INDICATOR				
STRATIFICATION INDICATOR	Long-standing illness	Overweight	Use of mental health services	Poor self-rated health	Weekly health complaints

	Cumulative odds ratios with 95% confidence intervals^2)^				

MODEL 1^1)^					

Cars in the family					
Two or more	1.0 (ref)	1.0 (ref)	1.0 (ref)	1.0 (ref)	1.0 (ref)
One	1.0 (0.8–1.2)	1.0 (0.8–1.1)	**1.3 (1.0–1.6)**	0.9 (0.8–1.0)	1.0 (0.9–1.1)
None	1.2 (0.9–1.6)	**1.4 (1.1–1.9)**	1.4 (0.9–2.1)	1.2 (1.0–1.5)	1.2 (1.0–1.5)

Vacation travels					
Three or more	1.0 (ref)	1.0 (ref)	1.0 (ref)	1.0 (ref)	1.0 (ref)
Two	0.9 (0.7–1.1)	**1.3 (1.0–1.6)**	1.0 (0.7–1.4)	**1.3 (1.1–1.5)**	1.0 (0.9–1.2)
One	0.9 (0.7–1.1)	1.1 (0.9–1.4)	1.3 (0.9–1.7)	**1.5 (1.3–1.7)**	1.1 (1.0–1.3)
None	1.0 (0.8–1.2)	**1.4 (1.1–1.7)**	**1.6 (1.2–2.2)**	**1.8 (1.5–2.1)**	**1.3 (1.1–1.5)**

Computers					
Three or more	1.0 (ref)	1.0 (ref)	1.0 (ref)	1.0 (ref)	1.0 (ref)
Two	0.9 (0.7–1.2)	0.9 (0.7–1.3)	0.8 (0.5–1.1)	1.0 (0.8–1.2)	1.0 (0.8–1.2)
One	0.9 (0.7–1.2)	1.1 (0.8–1.4)	0.9 (0.6–1.3)	**0.8 (0.7–1.0)**	0.9 (0.7–1.0)
None	1.1 (0.8–1.6)	1.1 (0.8–1.6)	1.3 (0.8–2.1)	1.2 (1.0–1.6)	1.1 (0.8–1.3)

Own room					
Yes	1.0 (ref)	1.0 (ref)	1.0 (ref)	1.0 (ref)	1.0 (ref)
No	0.9 (0.7–1.1)	0.9 (1.0–1.1)	1.0 (0.7–1.4)	1.0 (0.8–1.1)	1.0 (0.8–1.1)

Weekly spending money					
Above upper quartile	1.0 (ref)	1.0 (ref)	1.0 (ref)	1.0 (ref)	1.0 (ref)
Median – Upper quartile	0.8 (0.6–1.0)	1.0 (0.8–1.2)	0.9 (0.6–1.1)	1.0 (0.8–1.2)	1.0 (0.8–1.1)
Lower quartile – Median	**0.8 (0.6–1.0)**	0.9 (0.7–1.2)	**0.5 (0.4–0.8)**	0.9 (0.8–1.1)	0.9 (0.7–1.0)
Below lower quartile	0.8 (0.6–1.0)	**0.8 (0.6–1.0)**	0.8 (0.6–1.1)	0.9 (0.8–1.1)	0.9 (0.8–1.1)

MODEL 2 ^3)4)^					

Cars in the family	**. .**			**. .**	**. .**
Two or more		1.0 (ref)	1.0 (ref)		
One		1.0 (0.8–1.2)	**1.3 (1.0–1.6)**		
None		**1.4 (1.0–1.8)**	1.3 (0.9–2.0)		

Vacation travels	**. .**				
Three or more		1.0 (ref)	1.0 (ref)	1.0 (ref)	1.0 (ref)
One or two		1.2 (1.0–1.5)	1.1 (0.8–1.5)	**1.4 (1.2–1.6)**	1.1 (1.0–1.2)
None		**1.4 (1.1–1.7)**	**1.6 (1.2–2.2)**	**1.8 (1.5–2.1)**	**1.3 (1.1–1.5)**

Computers	**. .**	**. .**	**. .**		**. .**
Two or more				1.0 (ref)	
One				**0.8 (0.7–0.9)**	
None				1.2 (0.9–1.4)	

Own room	**. .**	**. .**	**. .**	**. .**	**. .**

Weekly spending money				**. .**	**. .**
Above upper quartile	1.0 (ref)	1.0 (ref)	1.0 (ref)		
Lower quartile -Upper quartile	**0.8 (0.6–1.0)**	1.0 (0.8–1.2)	**0.7 (0.5–0.9)**		
Below lower quartile	0.8 (0.6–1.0)	**0.8 (0.6–1.0)**	0.8 (0.6–1.1)		

^1) ^Models: Health variable = Stratification indicator + Sex/Age variable.

^2) ^Statistically significant cumulative odds ratios in bold.

^3) ^Models for stratification indicators independently associated with the health variable: Health variable = Stratification indicator_1 _+ Stratification indicator_2 _... +Stratification indicator_k _+ Sex/Age variable.

^4) ^The categories giving approximately equal odds ratios were combined.

**.. **= Not statistically significant in the previous step of analysis

**Table 4:** Ordinal logistic regression models for associations between personal social position and poor health. Separate models for each health variable, adjusted for sex and age

	HEALTH INDICATOR				
STRATIFICATION INDICATOR	Long-standing illness	Overweight	Use of mental health services	Poor self-rated health	Weekly health complaints

	Cumulative odds ratios with 95% confidence intervals^1)^				

MODELS 1–2^2)^					

School achievement^3)^					
Much above average	1.0 (ref)	1.0 (ref)	1.0 (ref)	1.0 (ref)	1.0 (ref)
Slightly above average	0.9 (0.7–1.1)	1.2 (0.9–1.5)	1.1 (0.8–1.7)	**1.5 (1.3–1.8)**	1.0 (0.9–1.2)
Average	0.8 (0.7–1.1)	**1.6 (1.2–2.1)**	1.4 (0.9–2.0)	**2.1 (1.8–2.4)**	**1.3 (1.1–1.5)**
Below average	1.2 (0.9–1.5)	**1.4 (1.1–1.9)**	**4.0 (2.7–5.9)**	**3.7 (3.0–4.5)**	**2.1 (1.7–2.5)**

^1) ^Statistically significant cumulative odds ratios in bold.

^2) ^Models: Health variable = School achievement + Sex/Age variable.

^3) ^Adolescents not in education or still in comprehensive school at age 16 were classified in the category "below average".

**Table 5:** Final ordinal logistic regression models^1)2) ^for associations between stratification indicators and poor health. Separate models for each health variable, adjusted for sex and age

	HEALTH INDICATOR				
STRATIFICATION INDICATOR	Long-standing illness	Overweight	Use of mental health services	Poor self-rated health	Weekly health complaints

	Cumulative odds ratios with 95% confidence intervals^3)^				

*Familial social position*					
Father's SES	**. .**	**. .**	*NS*	**. .**	**. .**
Higher					
Blue collar/Other					

Parent's education	***NS***		**. .**	*NS*	**. .**
Either has high level		1.0 (ref)			
Neither has high level		**1.5 (1.2–1.9)**			

Parent's labour market position	**. .**				
Both gainfully employed		1.0 (ref)	1.0 (ref)	1.0 (ref)	1.0 (ref)
At most one gainfully employed		**1.2 (1.0–1.4)**	**1.8 (1.4–2.3)**	**1.3 (1.2–1.5)**	**1.2 (1.0–1.3)**

*Family affluence*					

Cars in the family	**. .**	*NS*	*NS*	**. .**	**. .**

Vacation travels	**. .**	*NS*	*NS*		*NS*
Three or more				1.0 (ref)	
At most two				**1.4 (1.3–1.6)**	

Computers in the household	**. .**	**. .**	**. .**		**. .**
Two or more				1.0 (ref)	**. .**
At most one				**0.8 (0.7–0.9)**	

Own room	**. .**	**. .**	**. .**	**. .**	**. .**

Weekly spending money		*NS*	*NS*	**. .**	**. .**
Above upper quartile	1.0 (ref)				
At most upper quartile	**0.8 (0.6–1.0)**				

*Personal social position*					

School achievement^4)^					
Above average	**. .**	1.0 (ref)	1.0 (ref)	1.0 (ref)	1.0 (ref)
At most average		**1.3 (1.2–1.6)**	**1.7 (1.3–2.1)**	**1.7 (1.6–1.9)**	**1.4 (1.3–1.5)**

^1) ^Models for stratification indicators independently associated with the health variable: Health variable = Stratification indicator_1 _+ Stratification indicator_2_... + Stratification indicator_k _+ Sex/Age variable.

^2) ^The categories giving approximately equal odds ratios were combined.

^3) ^Statistically significant cumulative odds ratios in bold.

^4) ^Adolescents not in education or still in comprehensive school at age 16 were classified in the category "below average".

**.. **= Not statistically significant in the previous steps of analysis; *NS*= Not statistically significant in the model.
